# Blood Transfusion Practice before and after Implementation of Type and Screen Protocol in Emergency Department of a University Affiliated Hospital in Iran

**DOI:** 10.1155/2014/316463

**Published:** 2014-09-02

**Authors:** Mostafa Alavi-Moghaddam, Mahmoud Bardeh, Hossein Alimohammadi, Habib Emami, Seyed-Mostafa Hosseini-Zijoud

**Affiliations:** ^1^Department of Emergency Medicine, Imam Hossein Hospital, Shahid Beheshti University of Medical Sciences, Tehran, Iran; ^2^Department of Epidemiology, Masih Daneshvari Hospital, Shahid Beheshti University of Medical Sciences, Tehran, Iran; ^3^Social Development and Health Promotion Research Center, Kermanshah University of Medical Sciences, Kermanshah, Iran; ^4^Clinical Research Development Unit, Imam Hossein Hospital, Shahid Beheshti University of Medical Sciences, Tehran, Iran

## Abstract

*Background*. Blood transfusion is the cornerstone of therapy for many serious and common diseases. This study was performed to assess blood transfusion practice before and after implementation of type and screen protocol in emergency department of a university affiliated hospital in Iran, 2012-2013.* Methods*. An audit was studied before and after the implementation of type and screen protocol. The number of blood transfusions, time interval between blood order and transfusion, cross-match to transfusion ratio (*C*/*T* ratio), and transfusion index (TI) were checked.* C*/*T* ratio was used as a measure of the efficiency of blood ordering practice. We compared our results before and after implementation of type and screen protocol.* Results*. In present study after implementation of type and screen protocol, the time interval between requesting blood transfusion and transfusion of blood has decreased significantly (*P* < 0.001). The number of blood transfusions required by actual patients increased significantly from 1/2 to 2 (*P* < 0.001). The average cross-match to transfusion (*C*/*T*) ratio got near 1.13 from 1.41 and TI got near 0.91 from 0.58 (*P* < 0.001).* Conclusion*. The implementation of T&S protocol has been proven to be safe, efficient, and beneficial to the transfusion practice of our hospital from the current study.

## 1. Introduction

The requirements of pretransfusion assessment have undergone repeated modification, and the type and screen (T&S) protocol is currently the most widely accepted. The T&S protocol has been widely adopted by blood banks in North America and Europe in the mid-1980s [1]. The T&S protocol has been proven to be safe, efficient, and beneficial to the transfusion practice of hospitals [2, 3]. “Type” stands for the ABO and Rh typing, specifically the phenotype, and “screen” refers to testing for atypical antibodies that might cause transfusion problems. T&S protocol has been developed to prevent complications from incompatibility between donor and recipient due to the existence of irregular antibodies. This procedure is much cheaper than full cross-matching and gives the same immunohaematological safety [4, 5].

The efficiency of the T&S protocol may be measured by calculating the cross-match to transfusion (*C*/*T*) ratio. The more accurately the clinicians predict patient's blood needs, the closer the* C*/*T* ratio will be to 1 : 1. Thus a low* C*/*T* ratio signifies efficient hospital transfusion policy and practice [6].

Before this procedure became popular many units of donor blood were cross-matched and held in reserve for patients, who probably would not need it. At times this would cause shortage of blood supply and unnecessary outdating of donor units. These factors, along with the added expense of cross-matching blood, caused the T&S protocol to gain popularity. This procedure is most frequently used to screen preoperative or obstetrical patients whose risk of excessive blood loss is minimal. In case of an emergency, when blood is needed for these patients, cross-matched ABO and Rh compatible blood can be released with 99.9% assurance of safety, as long as the patient has no unexpected antibodies [6].

There has been concerns from doctors in our hospital as well as in different parts of the world [5, 7, 8] that excessive ordering of blood can lead to an unintentional misuse of blood bank services. It appears that physicians order cross-matched blood on the basis of habit or as part of hospital routines and there is a tendency in most emergency medical departments to order more units of blood than what are actually needed. The resulting unnecessary cross-matching is costly and wasteful.

This leads to strain on blood banks where the resources are limited. A review of blood ordering habits and blood utilization statistics in audit studies can help in improving these services and initiate measures to regulate blood ordering and utilization. However, a strong institutional commitment is required for implementation of new policies, albeit the advantages to this approach have been noted [3, 5, 6].

This policy has not attained a certain place in Iranian hospitals and blood banks in almost all hospitals in our country are still using the cross-matched and booking blood test before use. This study was performed to assess blood transfusion practice before and after implementation of the T&S protocol in the emergency department of a university affiliated hospital in Iran.

## 2. Methods

Imam Hossein Hospital is established in 1986 and is considered as a referral center in Iran. It is a 570-bed educational and general medical/surgical hospital and currently has wards of general surgery, internal medicine, pediatrics, gynecology and obstetrics, neurology, infectious, neurosurgery, ophthalmology, orthopedics, oncology, radiology, emergency, psychiatric, dialysis, and pathology as well as ICU, NICU, CCU, operation room, clinics, and clinical laboratory.

The blood transfusion chain data among patients admitted in emergency department of the hospital between July 2012 and August 2013 before and after implementation of the T&S protocol was analyzed. These study phases were including three months for pre-T&S and three months for post-T&S and six months for training of T&S protocol among emergency department staffs. The medical record numbers of the emergency ward patients were taken from the coded record and the data for cross-matched units and actual transfused units was retrieved from the blood bank database. The two data sets were merged using admission number as the unique primary key.

The present study assessed the blood transfusion practice in two phases, first phase for 472 patients before implementation and second phase for 472 patients after implementation of the T&S protocol in the emergency department of the hospital.

The number of blood transfusions, time interval between blood order and transfusion, the cross-match to transfusion ratio (*C*/*T* ratio), and transfusion index (TI) for each patient performed during the study period for both phases were calculated.* C*/*T* ratio is used as a measure of the efficiency of blood ordering practice. A ratio of more than 2.5 indicates excessive cross-matching of blood for a specific procedure. The transfusion index is defined as the average number of units transfused for a given procedure. TI of more than 0.5 indicates that blood needs to be cross-matched preoperatively for that procedure. However, TI has a potential of being influenced by occasional large transfusions [5, 6].

This study was approved by Ethics Committee of Shahid Beheshti University of Medical Sciences. Training workshop regarding the T&S protocol was given to blood bank technicians in the hospital by Iranian Blood Transfusion Organization experts. The T&S protocol consists of two stages: (1) performing complete ABO and Rh typing on the patient's blood sample and (2) screening the patient's serum for atypical antibodies [9].

## 3. **Statistical Analysis**

The statistical analysis was performed using SPSS version 18. Mean, median, and range of each variable are reported. The* C*/*T* and TI were calculated. Kolmogorov-Smirnov test was used for testing the normality of data before and after implementation of the T&S protocol.* t*-test was used to compare the quantitative data with normal distribution and Mann-Whitney test was used for data which were not normally distributed. For qualitative data chi-square test was used. Sample size was calculated to be 944 based on the transfused units (*α* = 5%, 1 − *β* = 90%, *P*1 = 60%, *P*2 = 70%, *D* = 10%, *n* = 472 in each phase of the study). A *P* value less than 0.05 was considered significant.

## 4. Results

This clinical audit of transfusion practices before and after implementation of the T&S protocol was conducted for 944 patients (472 in each phase) who had blood transfusion in emergency department of our hospital during 2012-2013. There were no statistically significant differences in mean age and BMI or the number of males (Table 1). In current study, the patients had suffered from trauma, GI bleeding, and cancers where this proportion did not change significantly in two phases (Table 1).

Blood transfusion data for patients is shown in Table 2. The actual number of patients who had blood transfusions increased significantly from 1.2 before implementation of the T&S policy to 1.9 after implementation of the T&S policy (*P* < 0.001).

The time interval between blood order and transfusion significantly was reduced from 143 min before to 26 min after the T&S protocol implementation.

The average cross-match to transfusion (*C*/*T*) ratio was reduced from 1.41 to 1.13 after the T&S protocol implementation, whereas the transfusion index (TI) increased from 0.58 to 0.91 after implementation of the T&S protocol.

## 5. Discussion

Many hospitals in developed countries have adopted the policy of using a T&S protocol instead of cross-match for transfusion practices. This technique has been proven to be effective without compromising patient safety [3, 5, 9, 10].

One of the best methods of evaluating transfusion practices is to determine the ratio of units cross-matched to units transfused (*C*/*T* ratio). The more accurately the clinicians predict patient's blood needs, the closer the* C*/*T* ratio will approach 1 : 1. Thus, a low* C*/*T* ratio signifies efficient hospital transfusion policy and practice [6, 11]. In the second phase of the current study, the T&S procedure has been proven to be effective in reducing unnecessary cross-matches for emergency department patients. The* C*/*T* ratio decreased from 1.41 to 1.13 since the implementation of the T&S protocol in emergency department of the hospital. There appear to be many causes for a high* C*/*T* ratio before the implementation of the T&S protocol, which includes outdated medicolegal habits or policies, lack of a clear blood ordering policy in hospitals, lack of clinical audits, and lack of communication between clinicians and blood bank health care workers.

Furthermore, the transfusion index (TI) increased from 0.58 to 0.91, after the T&S protocol implementation. TI is defined as the average number of units transfused for a given procedure. TI of more than 0.5 indicates that blood needs to be cross-matched preoperatively for that procedure.

The implementation of the T&S protocol provides many benefits to patients. After the T&S protocol implementation in the hospital the number of real blood transfusions increased significantly. This means that proper blood supplies were prepared for patients. The blood issued was safe and compatible. According to the conventional full cross-match test, blood units are randomly cross-matched without information about the patient's antibody status. The presence of weak antibodies may be missed if donor blood is heterozygous for an antigen. This deficiency is corrected by the T&S protocol, which stipulates that antibodies must be systematically screened using selected group-O reagent erythrocytes that harbour representative antigens. Patients who require a massive transfusion will benefit most from the T&S protocol, because as many additional compatible blood units as required can be issued quickly without the need for taking a new blood sample for repeating the cross-match. The problem of requiring maternal blood samples for repeated cross-matching (as previously required for a full cross-match test) now no longer exists. Previously, when the traditional full cross-match test was practiced, blood stock control was inflexible; thus, a blood unit could be reserved for a patient for only 3 to 4 days, depending on individual hospital policy [9, 10].

The time interval between blood order and blood transfusion was significantly reduced after the T&S protocol implementation. When the traditional full cross-match test was practiced in the hospital before implementation of the T&S protocol, blood units were reserved for a designated patient for two days. If the reserved units had been depleted or had exceeded the reservation date, repeated blood sampling and cross-matching would have been required if additional units were needed. This arrangement led to additional blood taking by the front-line clinicians. In addition, as a repeated cross-match required at least another one to two hours, depending on the full cross-match methodology used. There was a tendency to overestimate the number of blood units that would be needed. Hence, not only did the workload of the blood bank staff increase, but also the blood stock needed for emergency use was jeopardized. But under the T&S protocol blood units were no longer reserved for a patient if the results from the antibody screen were negative. Instead, a validity period is given to an individual for its negative antibody screen status, so that within such a period as many units as possible can be issued after performing an abbreviated cross-match depending on the amount of serum available. For patients who have received a transfusion or who have been pregnant within the preceding 3 months of the transfusion or whose history is unknown, the validity period given is 3 days, since antibodies developed within that time. Thus, for three consecutive days, no additional blood sampling is performed, even if repeated transfusions are required. In addition, as the abbreviated cross-match takes less than 10 minutes to perform, compatible blood units can be made available within a short time. Consequently, overordering of blood units does no longer exist [5, 9, 10].

These findings highlight the need for a T&S protocol implementation. Given the potential for using this method, it is necessary to implement this method and evaluate its benefits for the community. It is important to recognize that an effective implementation of such policy is dependent not only on the cooperation of all emergency medical and surgical specialties, but also on other related professions such as anesthetists and blood bank staff members. Hematologist very clearly should explain to users the process and pros and cons of the T&S protocol. In particular, it should be highlighted that in case of an emergency compatible blood can be provided within 5–7 minutes after doing a 2–5-minute rapid spin cross-match [9, 12].

## 6. Conclusion

In conclusion, although blood transfusion is a life saving measure for many patients, it should be restricted to patients who are in real need for blood replacement. Blood transfusion is safer today than it previously was due to the measures practiced to improve the quality of blood supply and to reduce the risk of transfusion-transmitted diseases. These include conservation of limited resources and costs and improved methods for blood ordering and utilization to limit wastage of blood and laboratory resources. The T&S protocol has been proven to be safe, efficient, and beneficial to the transfusion practice of a hospital. We suggest that all hospitals in developing countries that are currently experiencing a high* C*/*T* ratio and having a large workload of elective surgeries should consider adopting such a policy to allow better transfusion management.

## Figures and Tables

**Table 1:** Characteristics of patients in the pre- and postphases of implementation of the T&S policy.

	Before T&S	After T&S	*P* value
Age (years)	35.2 ± 7.3	38.3 ± 9.4	0.19
Sex (male/female)	391/81	387/85	0.71
Body mass index (kg/m^2^)	24.6 ± 4.7	25.3 ± 3.2	0.61
Trauma/GI bleeding/cancer (*n*)	197/183/92	189/187/96	0.52

Values are reported as the mean ± standard deviation.

**Table 2:** Blood transfusion data of 944 patients during the period of study.

	Before implementation of type and screen		After implementation of type and screen		*P* value
	Mean ± SD	Median (range)	Mean ± SD	Median (range)	
Transfusion number	1.2 ± 0.7	1 (0 to 5)	1.9 ± 0.7	2 (0 to 4)	<0.001
Time interval between order and transfusion	143 ± 160	104 (4 to 1344)	26 ± 110	8 (2 to 852)	<0.001
*C*/*T* ratio	1.41 ± 0.8	2 (0 to 4)	1.13 ± 0.4	1 (0 to 2)	<0.001
Transfusion index (TI)	0.58 ± 0.27	0.5 (0 to 1)	0.91 ± 0.21	1 (0 to 1)	<0.001

## References

[1] Klein HG, Anstee DJ (2008). *Mollison's Blood Transfusion in Clinical Medicine*.

[2] Chawla T, Kakepoto G (2001). An audit of blood cross-match ordering practices in The Aga Khan University Hospital: first step towards a maximum surgical blood ordering schedule. *Journal -Pakistan Medical Association*.

[3] Dexter F, Ledolter J, Davis E, Witkowski TA, Herman JH, Epstein RH (2012). Systematic criteria for type and screen based on procedure's probability of erythrocyte transfusion. *Anesthesiology*.

[4] Lin J-S, Chen Y-J, Tzeng C-H, Lyou J-Y, Lee C-H (2006). Revisiting of preoperative blood ordering policy—a single institute's experience in Taiwan. *Journal of the Chinese Medical Association*.

[5] Thabah R, Sailo LT, Bardoloi J (2013). 'Maximum surgical blood order schedule' in a newly set-up tertiary care hospital. *Anaesthesia, Pain and Intensive Care*.

[6] Chow E (1999). The impact of the type and screen test policy on hospital transfusion practice. *Hong Kong Medical Journal*.

[7] Juma T, Baraka A, Abu-Lisan M, Asfar SK (1990). Blood ordering habits for elective surgery: time for change. *Journal of the Royal Society of Medicine*.

[8] Gombotz H, Rehak PH, Shander A, Hofmann A (2007). Blood use in elective surgery: the Austrian benchmark study. *Transfusion*.

[9] Precautions A (2012). Transfusion of blood and blood products. *Atlas of Procedures in Neonatology*.

[10] Devbhandari MP, Farid S, Goatman C (2013). Is type and screen only policy safe for patients undergoing elective lobectomy?. *European Journal of Cardio-Thoracic Surgery*.

[11] Oliveira A, Fleming R, Galvão M (2006). The maximum surgical blood order schedule. *Acta Médica Portuguesa*.

[12] Palmer T, Wahr JA, O'Reilly M, Greenfield MLVH (2003). Reducing unnecessary cross-matching: a patient-specific blood ordering system is more accurate in predicting who will receive a blood transfusion than the maximum blood ordering system. *Anesthesia & Analgesia*.

